# Case of Absence of the Uterus

**Published:** 1850-11

**Authors:** Geo. Smith Crawford

**Affiliations:** Chicago


### ARTICLE IV.

*Case of Absence of the Uterus.*—By GEO. SMITH CRAWFORD' M. D., of Chicago.

EDITORS JOURNAL: Gentlemen,—If you think the enclosed worth publishing, it is at your service.

Sept. 19th. Mrs. A. D., æt. 30, has been married fourteen months, states that she never had any periodical discharge from the vagina, excepting about two years ago, when on one occasion there was a slight serous discharge, which she supposed to be the menstrual fluid; since girlhood she has had a small quantity of a whitish glairy fluid pass from the anus before the evacuation of fecal matter; about once a month, the discharge would continue for two or three days, on her going to stool; at those periods, she had pain in the loins, with general constitutional disturbance, and enlargement of the mammæ. Before marriage, she suffered much distress from pain and a sensation of fullness in the abdomen, not referable to any particular part; never was sensible of any pain in the region of the womb; since marriage, those anomalous feelings have not caused her much trouble; her general appearance at present is healthy, On examination, I could with difficulty introduce a small glass speculum, owing to unusual constriction of the sphincter; on withdrawing the speculum, which had not passed more than inch beyond the sphincter, I satisfied myself by a minute examination by the vagina and rectum, that the former terminated in a sac, and that there were no traces of a uterus.

Being desirous that my patient should be fully satisfied as to her condition, I appointed to call next day with a medical friend well versed in such matters, at which time Dr. Rutter was kind enough to attend, and, on examination, coincided with me as above; the depth of the vaginal sac does not exceed 1½ inches. She has sexual desires, and is gratified by [the attempt at] coition.

